# Sequestered defensive toxins in tetrapod vertebrates: principles, patterns, and prospects for future studies

**DOI:** 10.1007/s00049-012-0112-z

**Published:** 2012-08-04

**Authors:** Alan H. Savitzky, Akira Mori, Deborah A. Hutchinson, Ralph A. Saporito, Gordon M. Burghardt, Harvey B. Lillywhite, Jerrold Meinwald

**Affiliations:** 1 Department of Biology, Utah State University, Logan UT, 84322-5305 USA; 2 Department of Zoology, Graduate School of Science, Kyoto University, Sakyo, Kyoto, 606-8502 Japan; 3 Department of Biology, Coastal Carolina University, P.O. Box 261954, Conway, SC 29528 USA; 4 Department of Biology, John Carroll University, University Heights, Ohio, 44118 USA; 5 Department of Psychology, University of Tennessee, Knoxville, TN 37996-0900 USA; 6 Department of Zoology, University of Florida, Gainesville, FL 32611-8525 USA; 7 Department of Chemistry and Chemical Biology, Cornell University, Ithaca NY, 14853-1301 USA

**Keywords:** Sequestration, Dietary toxins, Antipredator defense, Aposematism, Amphibians, Reptiles

## Abstract

Chemical defenses are widespread among animals, and the compounds involved may be either synthesized from nontoxic precursors or sequestered from an environmental source. Defensive sequestration has been studied extensively among invertebrates, but relatively few examples have been documented among vertebrates. Nonetheless, the number of described cases of defensive sequestration in tetrapod vertebrates has increased recently and includes diverse lineages of amphibians and reptiles (including birds). The best-known examples involve poison frogs, but other examples include natricine snakes that sequester toxins from amphibians and two genera of insectivorous birds. Commonalities among these diverse taxa include the combination of consuming toxic prey and exhibiting some form of passive defense, such as aposematism, mimicry, or presumptive death-feigning. Some species exhibit passive sequestration, in which dietary toxins simply require an extended period of time to clear from the tissues, whereas other taxa exhibit morphological or physiological specializations that enhance the uptake, storage, and/or delivery of exogenous toxins. It remains uncertain whether any sequestered toxins of tetrapods bioaccumulate across multiple trophic levels, but multitrophic accumulation seems especially likely in cases involving consumption of phytophagous or mycophagous invertebrates and perhaps consumption of poison frogs by snakes. We predict that additional examples of defensive toxin sequestration in amphibians and reptiles will be revealed by collaborations between field biologists and natural product chemists. Candidates for future investigation include specialized predators on mites, social insects, slugs, and toxic amphibians. Comprehensive studies of the ecological, evolutionary, behavioral, and regulatory aspects of sequestration will require teams of ecologists, systematists, ethologists, physiologists, molecular biologists, and chemists. The widespread occurrence of sequestered defenses has important implications for the ecology, evolution, and conservation of amphibians and reptiles.

## Introduction

Both animals and plants are defended by an extraordinary array of molecules that render them noxious, and in some cases toxic, to potential predators. Although many of these compounds are synthesized by the defended taxa, others are acquired from environmental sources and redeployed in defense of the consumer. Examples are especially numerous among invertebrates, especially phytophagous insects (Opitz and Müller 2009) and certain groups of marine invertebrates, such as nudibranch mollusks (McPhail et al. 2001). In contrast, examples among tetrapod vertebrates are limited to a relatively small number of lineages. Indeed, although it has been nearly 20 years since John W. Daly documented their occurrence among Neotropical poison frogs (Dendrobatidae) (Daly et al. 1994a, b), sequestered toxins are still considered a novelty among vertebrates. Nonetheless since those early studies, reports have documented additional cases, some of them remarkably complex in their morphological, physiological, and evolutionary correlates. Here we review the known cases of toxin sequestration in tetrapods and discuss the broad ecological and evolutionary patterns discernable among those cases. Based upon commonalities that characterize these recognized cases, we suggest that the occurrence of sequestered defensive compounds (SDCs) in tetrapods may be more widespread, and the number of cases far more numerous, than has been appreciated to date.

We believe that the incidence of SDCs among ectothermal tetrapods (“amphibians” and “reptiles” as these terms are commonly applied) has been greatly underestimated, based on the evidence summarized below. In our opinion, two factors are responsible for the failure to appreciate fully the scope of SDCs among tetrapod vertebrates. First, few field researchers themselves have the expertise required to analyze diverse defensive compounds, to examine the morphological basis for the delivery of those compounds, or to study the physiological and behavioral effects of those compounds on potential predators. Second, the scattered observations that suggest the existence of SDCs among many amphibians and reptiles have, until recently, failed to engender discussion among researchers studying disparate taxa. Without an explicit conceptual framework, many relevant observations have lingered as isolated studies in the literature or simply as anecdotes shared informally among field researchers.

In this review, we attempt to remedy that situation by providing such a conceptual framework. We first review briefly the known cases of sequestration of defensive toxins in tetrapod vertebrates. Guided by these examples, we define our concept of sequestration and establish criteria for identifying potential cases of SDCs. We then discuss a number of potential cases of SDCs among amphibian and reptilian taxa. Finally, we address the implications of the widespread occurrence of SDCs and suggest a general research program for their further study. Whether or not our suspicions of sequestration by particular taxa are ultimately substantiated, it is clear that for many of those taxa the hypothesis that defensive toxins have an environmental source, or even that such toxins occur at all, has not been considered seriously, much less tested. Our goal is to encourage further studies of the nature and sources of defensive toxins in a wider range of tetrapod lineages by suggesting potentially fruitful avenues for future investigation.

## Known examples of sequestration in tetrapods

Among Tetrapoda, most examples of sequestration have been reported from ectothermal taxa, as detailed below. However, two genera of birds (*Pitohui* and *Ifrita*) are known to sequester prey toxins (Dumbacher et al. 1992, 2000), and a variety of other birds are suspected of being toxic (Berenbaum 1995; Dumbacher and Pruett-Jones 1996; Weldon and Rappole 1997), although the source of their suspected toxins is undetermined. Secreted chemical defenses among mammals are limited to the tarsal venom delivery system of monotremes and the musk glands of several taxa (Berenbaum 1995). The venom of certain insectivores is employed in predation, rather than defense (Greene 1997). For none of these mammals has an exogenous origin of the toxins been suggested, nor is it likely. It is certainly possible that chemical defense, including sequestration, has been underappreciated in both birds and mammals, as we suggest below for amphibians and non-avian reptiles. If so, the following discussion might provide a model for similar investigations of endothermic tetrapods. Conversely, it is possible that the differently tuned metabolic rates of ectotherms render them better able to evolve tolerance to exogenous toxins than are endotherms, and thus more likely to evolve defenses based on internal sequestration of environmental toxins. This could explain the prevalence among endotherms of self-anointing external body surfaces with toxins, rather than sequestration of exogenous toxins within internal tissues.

Among the several groups of ectotherms known to sequester prey toxins (Table 1), the earliest and still best-known examples involve the poison frogs, five lineages of anuran amphibians that have independently evolved the capacity to store lipophilic alkaloids obtained from dietary arthropods. The most extensively studied taxa are the Dendrobatidae, a family of Neotropical frogs that afforded the first documented examples of sequestration in tetrapods (Daly 1995). Many, but not all, members of the speciose Dendrobatidae sequester alkaloids, whereas members of its sister taxon, the Aromobatidae, apparently do not (Grant et al. 2006). The sources of dendrobatid toxins include ants (Jones et al. 1999b; Spande et al. 1999; Saporito et al. 2004), beetles (Dumbacher et al. 2004), and millipedes (Saporito et al. 2003). Recently, oribatid mites have been identified as an additional source of alkaloids (Saporito et al. 2007a), and this class of prey is likely to prove especially important given their abundance in the diets of these small frogs (see also Raspotnig et al. 2011; Vences et al. 2011). Many of the same prey classes are suspected of serving as sources of toxins for the other groups of poison frogs, which include the Malagasy *Mantella* (Mantellidae; Daly et al. 1997; Clark et al. 2005), the Australian *Pseudophryne* (Myobatrachidae; Daly 1998; Smith et al. 2002), the South American *Melanophryniscus* (Bufonidae; Daly et al. 2007), and the Cuban species *Eleutherodactylus iberia* and *E. orientalis* (Eleutherodactylidae; Rodríguez et al. 2011). Despite their five independent evolutionary origins (not counting the several independent origins of sequestration within the Dendrobatidae themselves; Grant et al. 2006), these taxa share several common attributes, notably small size and, for most taxa, highly conspicuous and presumably aposematic coloration (Vences et al. 1998). Their diminutive size renders them significant as predators on the small, chemically defended arthropods mentioned, and also leaves them vulnerable to diurnal, visually oriented predators such as birds.

**Table 1:** Classes of dietary toxins, their prey sources, and tetrapod taxa known or presumed to obtain such toxins from prey, as well as candidate taxa suspected of sequestering such toxins

Toxins	Prey taxa	Predators known or presumed to sequester toxins	Selected candidate taxa that may sequester toxins
Lipophilic alkaloids	Ants (several families), oribatid mites, coccinellid and choresine beetles, siphonotid millipedes	Dendrobatidae, Mantellidae, *Melanophryniscus*, *Pseudophryne*, *Eleutherodactylus*	*Hemisus*, *Myobatrachus*, *Rhinophrynus*, *Hemidactylium*, *Phrynosoma*, *Moloch*, Scolecophidia (ants); *Liophis* (dendrobatid frogs)
Tetrodotoxin	Salamandridae, *Atelopus*	*Thamnophis*	*Liophis*
Bufadienolides	Bufonidae	*Rhabdophis*	*Heterodon*, *Liophis*, *Lystophis*, *Waglerophis*, *Xenodon*
Terpenes	Termites, slugs	None confirmed	Scolecophidia (termites); *Contia*, *Sibon*, *Dipsas*, *Duberria*, *Storeria*, Pareatidae (slugs)

This list of candidate taxa is not comprehensive; see text for additional taxa suspected of sequestering these toxins and for relevant citations

A truly independent origin of sequestration in dendrobatids and *Melanophryniscus* may be illusory. A recent phylogeny (Grant et al. 2006) places *Melanophryniscus* at the base of the bufonid lineage, which in turn is sister to the Dendrobatoidea + Hylodidae (the combined lineage comprising the Agastorophrynia). Although members of the Hylodidae and Aromobatidae (which, with Dendrobatidae, comprise the Dendrobatoidea) are not known to be especially toxic, the relatively close relationship between Dendrobatidae and Bufonidae, and the basal position of *Melanophryniscus* within the latter, suggest that tolerance of prey alkaloids, if not sequestration itself, may have evolved in the common ancestor of Agastorophrynia and Bufonidae.

A more nuanced interpretation is provided by Santos and Grant (2011), who argue on phylogenetic grounds that diurnal activity evolved in the common ancestor of Agastorophrynia, putting its members in contact with chemically defended diurnal prey, as well as visually oriented predators. Their reconstruction also shows a reversal of diel activity to nocturnality in later bufonid clades, which are solely defended by synthesized bufadienolides. This, in turn, leads to the intriguing possibility that the more important ancestral source of toxicity among Bufonidae, notorious for their production of diverse and powerful cardiotonic steroids, involved sequestration rather than synthesis of integumentary toxins. The idea of sequestration as an evolutionary stepping stone to the synthesis of toxins deserves further attention, as does the role of phylogenetic proximity in providing a shared genetic substrate for the independent evolution of resistance to toxins.

A second known case of toxin sequestration involves populations of snakes in the genus *Thamnophis* (Colubridae: Natricinae) that occur in western North America and consume newts (*Taricha*; Salamandridae), whose skin contains tetrodotoxin (TTX), a potent neurotoxin widely distributed across diverse metazoan taxa (Mebs 2001). The past decade has seen an intense focus on the predator–prey interactions between three species of *Thamnophis* and several species of *Taricha*, including identification of the key mutations in the molecular target of TTX, the voltage-gated sodium channels of cellular membranes (Feldman et al. 2009). The best studied of these interactions, between *Thamnophis sirtalis* and *Taricha granulosa*, includes extensive geographic variation in both toxicity of prey and resistance of the predator to TTX, which covary in a manner that implies an ongoing “arms race” between the two taxa. Significantly, even highly resistant snakes are not completely immune to the effects of TTX. Ingestion of high levels of the toxin by such individuals incurs a performance cost, as measured by locomotor capacity (Brodie III et al. 2005). Although presumably impaired in their ability to escape from their own predators, *T. sirtalis* that have consumed newts are nonetheless protected themselves by the accumulation of TTX in their own tissues, especially the liver, at levels that would be aversive, if not lethal, to mammalian or avian predators (Williams et al. 2004). Remarkably, such snakes apparently can survive partial removal of the liver by predatory birds, so prolonged retention of TTX in that organ might afford protection from some predators. In highly resistant populations of *T. sirtalis*, locomotor performance improves relatively quickly (over several hours; Brodie III et al. 2002), whereas the level of TTX in the snake’s tissues declines gradually over a period of weeks to months (Williams et al. 2004, 2012). Furthermore, evidence suggests that snakes in populations that are likely to consume newts in large quantities are aposematically colored and less inclined to rely upon flight as a defense (Williams et al. 2012). Whether this example involves active accumulation of TTX in selected tissues, delayed clearance of the toxin from such tissues, or simply a normal pace of clearance of that virulent toxin remains to be determined. In any event, the greater risk of exposure of such snakes to their own predators appears to be countered by the presence of ingested TTX in their tissues, qualifying as an example of defensive toxin sequestration, albeit a conceptually simple one.

Recently a more complex example of toxin sequestration has been demonstrated in another natricine snake, the Asian species *Rhabdophis tigrinus*, in which evidence for defensive sequestration of toxins from dietary toads is unambiguous (Hutchinson et al. 2007). This species possesses a series of paired defensive structures, known as nuchal glands, in the skin of the neck. Studies in the 1980s showed that the glands contained bufadienolides, steroidal toxins similar to those of toads (reviewed by Mori et al. 2012). The diet of *R. tigrinus* consists primarily of anuran amphibians, including toads of the genus *Bufo*, leading Mori (2004) to suggest that these snakes may obtain their toxins from that dietary source. Experimental studies have since confirmed that the snakes ultimately obtain their toxins from toads that are consumed as prey (Hutchinson et al. 2007), although it also has been shown that hatchling snakes can emerge from the egg already imbued with toxins provisioned by their mother, if her own levels of bufadienolides are sufficiently high (Hutchinson et al. 2008, 2012).

Unlike the *Thamnophis*–*Taricha* system, chemical defense in *Rhabdophis* clearly involves more than simply tolerance and delayed clearance of an ingested toxin. The nuchal glands appear to function solely in the storage and delivery of dietary bufadienolides, and the snakes have evolved specific defensive behaviors that direct the glands toward an attacking predator (Mori et al. 2012). Although only *R. tigrinus* has been studied in detail, 12 additional species in three presumably related natricine genera possess nuchal glands. The glands of four species [*R. tigrinus*, *R. lateralis* (formerly considered a subspecies of *R. tigrinus*), *R. subminiatus*, and *R. nigrocinctus*] have been reported to contain bufadienolides (Mori et al. 2012; D. A. Hutchinson, A. Mori, A. H. Savitzky, and H. Ota, unpublished), but nuchal gland fluid from the other species in this lineage has not yet been studied. The diversity among these related taxa promises to reveal much about the origin of this most elaborate of vertebrate sequestration systems. It is likely that this system, like that of *Thamnophis*, owes its origin to a general tolerance of prey toxins documented among natricine snakes. *T. sirtalis* is known to survive high levels of force-fed bufadienolides (Licht and Low 1968), and *Tropidonophis*, the sole natricine genus in Australia, is the most highly resistant snake in that region to the integumentary secretions of the introduced cane toad, *Rhinella marina* (Phillips et al. 2003).

## What constitutes sequestration?

In light of these known examples, it is reasonable to ask what attributes define sequestration as a defensive adaptation. Like many biological phenomena, sequestration in its classic manifestation is easily recognized, but the boundaries of the phenomenon are more difficult to delineate. Diverse compounds are taken up through the gut or across the skin in vertebrates, and their degree of modification, tenure in different tissues, and biological functions vary considerably. Compounds might bioaccumulate in an organism’s tissues with detrimental effects, a circumstance especially common with heavy metals and organic pollutants such as pesticides (Walker et al. 2001). The accumulation of such anthropogenic pollutants mimics the uptake of sequestered compounds, but obviously has been of no previous evolutionary significance.

In a comprehensive review of sequestration among insects, Duffey (1980) noted that the ability to take up and retain compounds is a ubiquitous feature of living systems. He regarded all such instances of uptake as sequestration, sensu lato, distinguishing between “casual sequestration”, “uptake of nutrients”, and other examples falling under his broad definition. While not contesting his basic premise, we suggest that the term “sequestration,” if it is to be ecologically meaningful, should be more narrowly defined in the context of defense or other functions for which the condition evolved. *We therefore define sequestration as the evolved retention within tissues of specific compounds, not normally retained in the ancestors of the taxon in question, which confers a selective advantage through one or more particular functions*. Thus defined, sequestration can be analyzed within the conceptual framework of the historical analysis of adaptation (Greene 1986; Larson and Losos 1996).

This definition acknowledges that sequestration of exogenous compounds may play a role not only in defense, but also in courtship, sexual selection, morphogenesis, or other behaviors or physiological processes. In some cases the same compound can serve several of these functions, as documented among a number of insects. For example, the terpenoid cantharidin appears to be synthesized only by beetles of the families Meloidae and Oedemeridae, but it is consumed and sequestered by numerous unrelated beetles, as well as some hemipterans, flies, and wasps (Dettner 1997), which use the toxin for defense. Several pyrochroid beetles also employ sequestered cantharidin as a courtship pheromone and can transfer it to the female during mating as a nuptial gift, some of which is later incorporated into the eggs (Eisner et al. 1996a, b; Dettner 1997; Eisner 2003b). The North American arctiid moth *Utetheisa ornatrix* sequesters pyrrolizidine alkaloids from larval food plants, and the alkaloids serve a defensive function both in the adults and in eggs provisioned by females with the toxin. Males can transfer the alkaloid to females during mating via the spermatophore, elevating the female’s concentration of alkaloid and increasing the concentration in her eggs (González et al. 1999). Females select mates on the basis of males’ concentration of alkaloid, which also serves as the basis for the species’ courtship pheromone (Eisner 2003b). In the Asian arctiid moth *Creatonotos gangis*, which also sequesters pyrrolizidine alkaloids for defense and courtship, the alkaloids additionally play a role in morphogenesis of the coremata, the brushlike organs that deliver the pheromone. The size of the coremata correlates with the concentration of alkaloids in the male (Schneider et al. 1982). To date only the defensive function of sequestered compounds has been studied among tetrapod vertebrates, but the possibility that such compounds serve additional functions, perhaps as pheromones or morphogens, deserves attention.

Even under this narrower definition, defensive sequestration spans a continuum from simple accumulation of toxins in unmodified tissues to the evolution of specialized delivery systems. Following the initial evolution of physiological tolerance to the toxins in question, a logical prerequisite, the evolution of sequestration can lead to several manifestations, which can be arrayed along a phenotypic spectrum from least to most complex. The first is simply accumulation of toxins in the consumer’s tissues, possibly involving differential accumulation in various tissue compartments. Among tetrapod vertebrates, this condition apparently is exemplified by *T. sirtalis*, which especially accumulates TTX in the liver and kidneys (Williams et al. 2004). A more highly derived condition involves the active concentration of toxins, perhaps involving the hypertrophy of pre-existing structures. This condition exists in dendrobatid frogs, in which toxins present in minute quantities in the prey attain high concentrations in the frogs’ skin, which has an unusually dense array of granular glands (Saporito et al. 2010). The final stage involves evolution of altogether novel structures for the storage and/or delivery of the sequestered toxins, as in *Rhabdophis* (Hutchinson et al. 2007; Mori et al. 2012). Our concept of defensive sequestration, as the evolved capacity for accumulation of environmental toxins with a concomitant advantage in antipredator defense, encompasses all three steps in this phenocline.

We also recognize a continuum in the degree of modification to which sequestered compounds may be subjected during or subsequent to uptake, and we accept some modest modifications under our definition. In many cases sequestered molecules are taken up and stored intact, as in the accumulation of TTX by *T. sirtalis* (Williams et al. 2012) and, apparently, the acquisition of most poison frog alkaloids (Saporito et al. 2012). Some dendrobatid frogs, however, can hydroxylate a specific dietary pumiliotoxin to a more toxic allopumiliotoxin (Daly et al. 2003). Similarly, *R. tigrinus* can modify some ingested bufadienolides through hydrolytic cleavage, hydroxylation, and/or epimerization (Hutchinson et al. 2012). Such modification has parallels among herbivorous insects. Pyrrolizidine alkaloids are stored in most of the plants as nontoxic N-oxides, but the compounds generally are converted to toxic free bases in the gut of herbivores (Hartmann et al. 2005b). The chrysomelid beetle *Platyphora* avoids toxicity by transferring toxic free bases effectively to specialized exocrine glands, where they are isolated from other tissues. In contrast, the related genus *Oreina* suppresses reduction of N-oxides in the gut, and the nontoxic N-oxides are accumulated in both the exocrine glands and the hemolymph (Hartmann et al. 2003). Arctiid moths also detoxify the free bases of pyrrolizidine alkaloids, by converting them back to N-oxides for storage (Hartmann et al. 2005a, b). Whether the modification of toxins documented in vertebrates is similarly related to the imperatives of uptake, detoxification, and storage remains to be determined. In any event, we consider such cases of limited modification to constitute sequestration, inasmuch as the ingested toxin is essential to production of the ultimate compound and the fundamental chemical structure (and in many cases the basic defensive function) of the precursor molecule is retained. In some cases, however, ingested compounds are fundamentally altered in structure, as with the extensive modification of some ingested pyrrolizidine alkaloids into new classes of insect alkaloids in both chrysomelids and arctiids (Hartmann et al. 2003, 2005a) or into sexual pheromones in some lepidopterans (Eisner 2003b). In such cases, we would consider the derivative molecules to be the result of extensive synthesis, albeit based upon essential sequestered precursors.

Our definition excludes from sequestration two related but distinct phenomena. Anointing behavior involves the application of defensive chemicals to exposed surfaces of the body (Weldon and Carroll 2007; Kingdon et al. 2011), rather than uptake and incorporation of exogenous compounds into the predator’s tissues. Some mammals anoint themselves with toxins or odorants as a defense against predators (Brodie Jr 1977a; Clucas et al. 2008; Kingdon et al. 2011), and both mammals and birds anoint themselves with arthropod secretions, apparently to deter parasites (Weldon and Carroll 2007). Similar application of compounds to external surfaces occurs among arthropods. For example, some slave-making ants and myrmecophilous beetles are defended by chemical mimicry or camouflage. The chemical cues may be adsorbed through physical contact with the prey or host species or by application during grooming (Dettner and Liepert 1994; Lenoir et al. 2001; Tsuneoka and Akino 2012).

Similarly, the production of antibiotic compounds by microbial ectosymbionts (Brucker et al. 2008a, b) is excluded from our definition, inasmuch as both the symbionts and their antimicrobial or antimycotic products appear to be limited to external surfaces (although they may transit occasionally through the gut; Wiggins et al. 2011). Nonetheless, although our concept of sequestration is intended to recognize chemical defenses directed against predators, it is broad enough to include exogenous compounds that offer defense against pathogens and parasites, if such compounds ultimately reside within the animal’s tissues.

## Common themes and candidate taxa

Based on the known cases of defensive toxin sequestration in both invertebrates and vertebrates, we can recognize two general attributes that, together, characterize most known sequestering taxa. First, such taxa generally consume a diet rich in compounds that are toxic to most other animals. In addition, such taxa generally exhibit defensive behavior that involves some form of relatively passive defense, whether immobility, aposematism, and/or mimicry. Although the first criterion (consumption of toxic prey) seems both obvious and essential for sequestration, in fact animals might sequester toxins from symbiotic or infectious microorganisms, as in the case of TTX in tetraodontiform fishes (Williams 2010), rather than a dietary source. This possibility has not yet been demonstrated for any tetrapod. The second criterion (passive defense) simply serves as an indicator that the predator itself might be noxious, and does not by itself distinguish predators that sequester toxins from species that synthesize them. A similar suite of morphological and behavioral characteristics in insects has been termed a “chemical defense syndrome” (Whitman et al. 1985). Although such behavioral and physical traits are shared with many taxa that synthesize defensive toxins, species that combine such defensive attributes with a diet rich in toxic prey may reasonably be suspected of sequestering consumed toxins. The frequency with which these two attributes, toxic diet and passive defense, occur together among known sequestering taxa suggests that their co-occurrence can be used to identify additional species that *might* sequester dietary compounds and that therefore merit further study. We note, however, that the absence of aposematism does not necessarily rule out sequestration. Crypsis is seen, for example, in some insect larvae that sequester toxic phytochemicals (Lindstedt et al. 2011) and in some chemically defended nudibranch mollusks (Cimino and Ghiselin 2009). Indeed, some amphibians combine cryptic dorsal pattern with aposematic ventral coloration exposed under stress by the unken reflex (Brodie Jr 1977b; Grant et al. 2012).

In suggesting fruitful avenues for the discovery of additional examples of defensive sequestration among tetrapods, we have grouped potential target taxa—those likely to employ SDCs—into three broad categories based upon their prey and/or the toxins they possess (Table 1). The first group includes *predators on toxic arthropods*. Among such prey, ants have figured prominently in discussions of sequestration, as they contain a wide variety of defensive and signaling chemicals (Hölldobler and Wilson 1990), among them alkaloids. As noted, however, such compounds also occur in certain millipedes, beetles, and especially mites (Daly et al. 2002; Saporito et al. 2007a, 2012). It is possible that some of these arthropod prey taxa, in turn, sequester their alkaloids from dietary plants or fungi. Among the compounds that may be sequestered by arthropod prey themselves are nicotine (Saporito et al. 2012) and epibatidine, a nicotine-like alkaloid found in certain populations of the dendrobatid genus *Epipedobates* (Daly 2003). Furthermore, the plant alkaloids calycanthine, chimonanthine, and noranabasamine have been found in skin extracts of the dendrobatid *Phyllobates terribilis* (Tokuyama and Daly 1983). Like ants, termites are social and produce a wide variety of defensive compounds, notably terpenes (Pasteels et al. 1983; Prestwich 1988; Quintana et al. 2003). Despite the phylogenetic distance and chemical differences between ants and termites, the morphological adaptations for feeding on small, highly concentrated prey sometimes results in predatory lineages whose members consume either or both prey taxa, as in the case of scolecophidian snakes (Greene 1997).

The second category of target taxa includes *predators on toxic mollusks*. Slugs have been shown to be defended by a diterpene toxin (Schroeder et al. 1999), and although no examples of sequestration of slug toxins have been confirmed by chemical analysis, circumstantial evidence suggests that some taxa might sequester these compounds, as discussed below. The defensive behaviors of some snakes that feed on shelled mollusks also suggest that such prey may be a source of dietary toxins.

The third broad category includes *predators on toxic amphibians*. As noted, many newts and several frogs possess tetrodotoxin, toads produce bufadienolide steroids (BDs), and dendrobatid poison frogs possess an array of alkaloids derived from dietary arthropods (Erspamer 1994). Each of these amphibian taxa is subject to predation by at least one lineage of snakes (Myers et al. 1978; Feldman et al. 2012; Mori et al. 2012). We discuss the evidence for toxin sequestration by these three classes of predators below, reviewing known cases and suggesting taxa that should be considered candidates for future studies.

A fourth category of sequestering tetrapods may be warranted: amphibians defended by tetrodotoxin. Many species of newts (Salamandridae, including *Taricha*) contain TTX in their tissues, as do a number of anurans, such as *Brachycephalus* (Brachycephalidae), many species of *Atelopus* (Bufonidae), and some *Colostethus* (Aromobatidae) and *Polypedates* (Rhacophoridae) (Hanifin 2010). However, the source of TTX in these species remains uncertain. Diverse marine taxa, such as tetraodontiform fishes, apparently sequester tetrodotoxin from either their diet or a non-dietary microbial source (Williams 2010). A similar origin has been suggested for some terrestrial taxa (Mebs 2001), but whether TTX is synthesized by any or all of these amphibian species or is accumulated from a microbial source remains controversial (Hanifin et al. 2002; Lehman et al. 2004; Chau et al. 2011). If documented for any amphibians, this would constitute not only an additional class of sequestration but also the only one in which the toxin has a microbial origin. Furthermore, if TTX in *Taricha* has an exogenous origin, then sequestration of newt toxins by *Thamnophis* would constitute a case of secondary bioaccumulation.

Regardless of the source of TTX, it clearly resides within integumentary and other tissues of these vertebrates, in contrast to the antibiotics released by microbial ectosymbionts onto the surface of amphibian skin (Brucker et al. 2008a, b). The occurrence of two other guanidinium toxins (the TTX analog chiriquitoxin and the saxitoxin analog zetekitoxin), each of which occurs in one or more species of *Atelopus*, further complicates our understanding of this class of defensive compounds (Daly 2004).

### Predators on social insects and other toxic arthropods

Both amphibians and reptiles include lineages specialized as predators on social insects, either ants (a known source of alkaloids) or termites (a potential source of terpenes), and some species prey on other groups of toxic invertebrates. A wide array of toxic compounds is produced by both ants (Hermann and Blum 1981; Piek 1986; Hölldobler and Wilson 1990; Jones et al. 1990, 1996, 1999a) and termites (Eisner et al. 1976; Meinwald et al. 1978; Prestwich 1979; Baker et al. 1981, 1982; Deligne et al. 1981; Pasteels et al. 1983; Nagnan and Clement 1990; Pearce 1997).

The five known lineages of poison frogs obtain their toxins from a diversity of arthropod prey, but ants and mites appear to be especially important as sources of defensive alkaloids. As documented by the recent discovery of sequestration in certain diminutive Cuban *Eleutherodactylus* (Rodríguez et al. 2011), additional examples likely remain to be discovered. All of the known poison frogs are small, active foragers, such as dendrobatids and *Mantella*. Additional candidate species exist among the many anurans that exhibit a combination of small size, diurnal foraging habits, and aposematic and/or static defensive displays or reduced escape behaviors (Cooper et al. 2009). Among such taxa is *Rhinoderma*, which occurs in Chile and Argentina. Although noted for its highly cryptic dorsal pattern and shape, with a fleshy proboscis that enhances its mimicry of a fallen leaf, a common defensive behavior of this diurnal species involves flipping over and lying motionless on its back, exposing a bold black and white ventral pattern (Crump 2000). Detailed dietary information on *Rhinoderma* is lacking. Interestingly, although formerly recognized as its own monotypic family, recent phylogenetic analysis places *Rhinoderma* in the Cycloramphidae, a family not distantly related to the Dendrobatoidea (Grant et al. 2006). No doubt other small leaf-litter anurans on many continents are similarly attractive as candidates for sequestering toxins either from ants, mites, or other arthropods.

Certain members of the Microhylidae also specialize on ants (Wells 2007), and evidence suggests that their chemical interactions with both potential predators and commensals are substantial. The North American *Gastrophryne olivacea* is aversive to a range of potential vertebrate predators, including snapping turtles (*Chelydra*) and herons (*Ardea*) (Garton and Mushinsky 1979), although the nature of the defensive compounds is unknown. That species and several Neotropical and Asian microhylids are known to live in close association with theraphosid spiders, sharing the spiders’ burrows or tree holes and not subject to consumption by the spiders (Hunt 1980; Cocroft and Hambler 1989; Siliwal and Ravichandran 2008; Karunarathna and Amarasinghe 2009). Whether such behavior is mediated by chemical communication (allomones) and whether any such compounds have a dietary origin are unknown.

In addition to such diminutive, actively foraging species, there exists a phylogenetically heterogeneous but morphologically convergent group of larger anurans specialized for feeding on aggregations of social insects. These taxa have evolved stout bodies, short limbs, pointed snouts, and distinctive protrusible tongues (Nishikawa et al. 1999), and they presumably feed by rapidly consuming ants and/or termites concentrated at nests or along trails. Significantly, many of these species are brightly colored, and their habitus suggests that their capacity for locomotory escape from predators is limited. Among these taxa are *Rhinophrynus* (Rhinophrynidae; Lee 1996) of Middle America, *Hemisus* (Hemisotidae; Nishikawa et al. 1999) of Africa, and *Myobatrachus* (Myobatrachidae; Cogger 1992) of Australia, all of which feed exclusively on ants and/or termites. The recently described Indian genus *Nasikabatrachus* (Sooglossidae) also is reported to consume termites (Radhakrishnan et al. 2007), although the dorsum in this genus is uniformly dark gray or purple.

In addition to anurans, many small salamanders might consume toxic arthropods, especially mites, and might employ dietary toxins in their defense. Unfortunately, the diets and defensive chemistry of small salamanders are even less well understood than those of anurans. However, *Hemidactylium* (Plethodontidae) is a strong candidate for sequestration. This monotypic genus exhibits cryptic dorsal coloration, but has a bold black and white ventral pattern that is exposed during a static defensive display (Brodie Jr et al. 1974), similar to the defensive behavior of *Rhinoderma*. Both the skin and eggs of *Hemidactylium* are noxious or unpalatable to certain predators (Hess and Harris 2000), but the toxins have not been characterized. Also of potential interest are the diminutive (but cryptic) species in the genus *Thorius* (Plethodontidae), which are among the smallest known tetrapods (Hanken 1985).

The chemical ecology of caecilians (Gymnophiona) is even more poorly known. Caecilians possess a highly glandular integument (Jared et al. 1999), and the skin secretion of the South American genus *Siphonops* has cardiotoxic properties (Schwartz et al. 1999). Unfortunately, neither the identity of caecilian toxins nor their source (i.e., whether synthesized or sequestered) is known. Although earthworms generally are considered the common prey of caecilians (Savage 2002), the Indian species *Gegeneophis ramaswamii* (Caeciliidae) is reported to consume large numbers of both ants and termites, as well as other invertebrates (Measey et al. 2004). Indeed, although oligochaetes represented greater prey mass, social insects greatly dominated the diet of that species in terms of numbers, accounting for over 80 % of prey found in digestive tracts. The African caeciliid *Boulengerula taitana* also exhibits a strong preference for termites (Gaborieau and Measey 2004), and it is not known how many other caecilians have diets similarly rich in social insects. Although many caecilians are fossorial and exhibit little color or pattern, the African caecilian *Schistometopum thomense* is brilliantly colored (Nussbaum and Wilkinson 1989), suggesting aposematism. Although the diet of this species consists overwhelmingly of oligochaetes, small numbers of oribatid mites were recovered from digestive tracts (Delêtre and Measey 2004). Clearly much remains to be learned about both the chemistry of caecilian toxins and their origins.

Some reptiles also are specialized predators on social insects, and many have attributes that suggest possible sequestration of toxins from prey. Among lizards, species of *Phrynosoma* (Phrynosomatidae) and *Moloch* (Agamidae), in North America and Australia, respectively, are perhaps the most specialized myrmecophagous taxa (Pianka and Parker 1975; Pianka and Pianka 2000). Members of both genera are stout and short-legged, relying largely on crypsis for defense. However, several species of *Phrynosoma* defend themselves by squirting blood from the orbital sinus, employing a unique sphincter to pressurize the vessel (Sherbrooke and Middendorf 2001). Canid predators exhibit what appears to be taste aversion to the blood (Sherbrooke and Middendorf 2004), which is suspected of harboring toxins from ants consumed as prey.

Among geckos, the Australian genus *Diplodactylus* (Diplodactylidae) includes a distinctive, monophyletic subgenus *Strophurus*, members of which possess caudal glands that release a sticky, noxious defensive secretion (Rosenberg and Russell 1980; Richardson and Hinchliffe 1983). Many of these geckos also are brightly colored, and some have a defensive display in which the mouth is opened to reveal the brightly colored mucosa (Melville et al. 2004). The secretion of the caudal glands has not been characterized chemically, but some species in this subgenus feed largely on termites and others on beetles, which constitute another potential source of toxins (Pianka and Pianka 1976; How et al. 1986). Significantly, this subgenus is diurnal (Melville et al. 2004), which is unusual for geckos and presents a striking parallel to the diel behavior of poison frogs. A number of other geckos prey on termites (Pianka and Vitt 2003). The Neotropical Sphaerodactylini, which include some of the smallest known amniotes (with snout-vent lengths <20 mm), consume a diet of mites and other minute arthropods, and many are diurnal and brightly colored (Pianka and Vitt 2003; Gamble et al. 2011).

The South American iguanian lizard *Liolaemus monticola* (Liolaemidae) is a specialized predator on ants of the genus *Camponotus* (Jaksic et al. 1979), in contrast to its congeners, which are trophic generalists. *L. monticola* also differs from congeners in having an unusually small liver, a condition that has been interpreted as reflecting a reduced need to detoxify defensive compounds of prey, either due to higher resistance or to the sequestration, rather than detoxification, of such compounds (Jaksic et al. 1979).

Specialized predators on social insects also occur among snakes. The most basal split in the phylogeny of living snakes separates the majority of species, the Alethinophidia, from a distinctive group of highly fossorial and poorly known taxa, the Scolecophidia. Recent phylogenetic studies have greatly expanded our knowledge of the taxonomic diversity and historical biogeography of the Scolecophidia (Adalsteinsson et al. 2009; Vidal et al. 2010), but their basic ecology remains poorly understood. The Scolecophidia include five families, which may have evolved simultaneously with the early radiation of their primary prey, ants and termites (Vidal et al. 2010). Some scolecophidians are known to follow the pheromone trails of ants and termites (Gehlbach et al. 1971; Webb and Shine 1992), and individual snakes can consume up to hundreds or thousands of prey (primarily larvae and pupae) at one time, aided by highly unusual cranial kinesis (Kley 2001), after entering the nests of these social insects (Shine and Webb 1990; Webb et al. 2000, 2001).

The ability of scolecophidians to enter and feed within ant nests suggests that they may be chemically defended against the ants themselves, a situation that may be functionally similar to the spider *Cosmophasis*, which pre-empts ant alkaloids for chemical mimicry to prey upon ants undetected (Elgar and Allan 2004). Scolecophidians are unusual among snakes in possessing integumentary glands beneath the scales of the head (known, with little justification, as sebaceous glands; McDowell 1974), the secretion of which has not been characterized chemically. Scolecophidians also possess cloacal glands, and in *Leptotyphlops dulcis* the contents of those glands are repellant to ants (Watkins et al. 1969). In response to an attack by ants, *Leptotyphlops* writhes for up to several minutes, smearing the scales with what appears to be fluid from the cloacal glands, after which the ants are deterred from further attacks. A variety of free fatty acids has been reported from the cloacal glands (Weldon et al. 2008), but the contents of neither those nor the integumentary glands have been examined for the presence of defensive compounds sequestered from prey. The eggs of scolecophidians are sometimes laid within the nests of ants or termites and may be attended by the female (Greene 1997; Bruner et al. 2012), suggesting that embryonic provisioning with prey compounds may contribute to defense of the eggs.

There is also evidence that potential vertebrate predators may avoid eating scolecophidians. *L. dulcis* is reported to live in close association with screech owls (*Otis asio*), occupying the nest cavity and apparently feeding on both ants and the larvae of dipterans that are attracted to the nest (Gehlbach and Baldridge 1987). The apparent immunity of the snakes to predation by the owls suggests a possible chemical defense. Human disturbance of *L. dulcis* elicited similar smearing of the body with cloacal fluid, as well as slow locomotion and putative death-feigning behavior. Several were rejected as prey by predatory snakes (Gehlbach 1970).

### Predators on toxic mollusks

The discovery that a terrestrial slug is defended by a novel terpene toxin (Schroeder et al. 1999) suggests an explanation for the unusual defensive behavior of a number of slug-eating snakes. Like their marine counterparts, the nudibranchs, terrestrial slugs appear to have evolved toxicity as a substitute for the defense provided by a calcified shell. Indeed, nudibranchs are well known for sequestering defensive toxins from sponges and other sources (Cimino and Ghiselin 2009; Haber et al. 2010), as well as storing the untriggered stinging cells of cnidarians (Greenwood and Mariscal 1984; Greenwood et al. 2004). Unlike the marine species, there has been little chemical sampling of terrestrial slugs, but widespread chemical defense in this group appears plausible, if not likely.

Several lineages of snakes prey largely or exclusively on slugs and, in some cases, also shelled mollusks (snails). Of these, the most specialized are the Neotropical Dipsadini (sensu stricto), a group of five xenodontine colubrid genera, and the Asian Pareatidae, a lineage that branches early within the colubroid snakes. Although independently evolved, these two lineages share several unusual morphological features generally associated with the extraction of the soft parts from the shells of snails (Savitzky 1983; Hoso et al. 2007). Many of the species additionally, if not primarily, consume slugs. Little is known of the defensive behavior of pareatids, but many Dipsadini exhibit static defensive displays, notably assuming a conical posture, with the forepart of the body elevated (Cadle and Myers 2003). The unrelated xenodontine *Contia*, a specialized predator on slugs, has a contrasting black and white ventral pattern that is exposed when the snake elevates its body in a static defensive display (Leonard and Stebbins 1999). Juveniles also display that pattern when they lie on their backs and coil their bodies in response to a predator (Ovaska and Engelstoft 1999). The juvenile posture has been hypothesized to mimic toxic millipedes (Leonard and Stebbins 1999), but that interpretation is inconsistent with the adult behavior. Another slug specialist, the unrelated African *Duberria* (Lamprophiidae; Pyron et al. 2011), also assumes an unusual, static defensive posture (Branch 1998). Among the two most widespread species of *Storeria* (Colubridae: Natricinae), *S. occipitomaculata* specializes on slugs; it has a bright red venter, exposed during putative “death-feigning” (Jordan 1970), and flares its lips in response to a predator (do Amaral 1999). In contrast, *S. dekayi* preys primarily on worms and has a white venter and no obvious defensive behavior. Whether any of these specialized molluscivores employs sequestered prey toxins for their defense is not known, but the question clearly is worth pursuing.

### Predators on toxic amphibians

Two genera of natricine snakes, *Thamnophis* and *Rhabdophis*, are known to sequester dietary toxins from amphibian prey, specifically newts and toads, respectively. However, several additional lineages of snakes are known to feed on newts, toads, or other toxic amphibians, and those taxa have not yet been investigated for the presence of sequestered defensive toxins.

It is known that *Thamnophis* spp. that consume newts are resistant to the effects of tetrodotoxin by virtue of mutations in the sodium channel proteins that are the molecular target of TTX (Geffeney et al. 2002; Brodie III et al. 2005). Several other snakes also prey on amphibians defended by TTX (Feldman et al. 2012), including the natricine *Amphiesma pryeri* of Okinawa, which preys on the newt *Cynops ensicauda* (Mori and Moriguchi 1988). *Rhabdophis lateralis*, a mainland Asian species formerly considered conspecific with *R. tigrinus* of Japan (Takeuchi et al. 2012), consumes *Polypedates leucomystax*, a frog defended by TTX (Feldman et al. 2012). Whether *R. lateralis*, which possesses nuchal glands, sequesters TTX in those structures or in any other tissues is unknown. In Japan, some populations of *Gloydius blomhoffii* (Viperidae) consume newts in large numbers (up to 12.1 % of the diet; Central Research Laboratories 1999), although it is not known whether consumption of newts has any effect on the defensive behavior of this venomous species.

Bufophagous (toad-eating) species are even more widespread among snakes. Most of them are prey specialists, and many of those exhibit unusual defensive behaviors. The best-studied species belong to the North American xenodontine colubrid genus *Heterodon*, species of which primarily consume toads (Platt 1969). The same group is noted for its elaborate defensive repertoire, which includes hood-spreading, false strikes, and presumptive death-feigning (Greene 1988). In the latter behavior, the individual lies on its back and exposes its ventral surface, which in *H. nasicus* is contrastingly patterned. Presumptive aposematic and mimetic behaviors also are known in the independently evolved South American xenodontine genus *Lystrophis* (Yanosky and Chani 1988; Baptista de Oliveira et al. 2000), and several other bufophagous taxa engage specifically in “death-feigning,” including *Natrix natrix* (Colubridae: Natricinae; Gregory et al. 2007; Gregory 2008) and, most remarkably, the spitting cobra *Hemachatus* (Elapidae; Rasmussen et al. 2009), which clearly has other effective defensive behaviors at its disposal.

We suggest that, rather than truly feigning death, such behavior may serve to slow the attack by a predator, as documented in spider–insect interactions (Miyatake et al. 2004), and initiate investigative behavior that would reveal noxious chemical defenses based on toxins sequestered from toads. Indeed, “death-feigning” has been reported in *R. tigrinus* (Fukada 1961; Mutoh 1983). Although the nuchal glands of *R. tigrinus*, and presumably those of related genera, are specialized for the storage and delivery of sequestered toad toxins, bufophagous snakes that lack nuchal glands have never been investigated to determine whether bufadienolides are present in any of their tissues. Furthermore, although the presence of “death-feigning” behavior in naïve hatchling *Heterodon platirhinos* (Burghardt and Greene 1988) initially appears inconsistent with the hypothesis of sequestered dietary toxins, the discovery that female *R. tigrinus* can provision their embryos with dietary bufadienolides (Hutchinson et al. 2008) suggests that unfed hatchlings of other bufophagous snakes might similarly be defended chemically from the moment of hatching.

The South American xenodontine colubrid tribe Xenodontini contains a number of taxa that prey on highly toxic amphibians, including the bufophagous *Xenodon*, *Waglerophis*, and *Lystrophis* (*Xenodon* sensu lato; Zaher et al. 2009). Among the members of this tribe is a remarkable species, *Liophis epinephalus* of lower Central and northern South America, which feeds on anuran amphibians (Savage 2002) and is known to survive ingestion of three different classes of potent defensive toxins (Myers et al. 1978): bufadienolide steroids from toads and *Atelopus*, TTX and zetekitoxin from *Atelopus*, and alkaloids from *Dendrobates* and *Phyllobates* (including the potent homobatrachotoxin, of arthropod origin; Dumbacher et al. 2004). *L. epinephalus* exhibits a conspicuous defensive display that involves flattening the body, exposing brightly colored skin between the scales (Greene 1997; Savage 2002). Whether any or all of these toxins from its varied anuran prey are sequestered has yet to be determined. Unfortunately, populations of this snake may be declining along with its anuran prey, populations of which have succumbed to infection by the epizootic chytrid fungus (La Marca et al. 2005; Whiles et al. 2006).

## Broader implications of SDCs in tetrapod vertebrates

If our premise proves correct and sequestered defensive toxins are more widespread among tetrapods than is presently known, the ecological implications would be far-reaching. Conceptually, toxin sequestration strengthens the linkage between predators (sensu lato, including herbivores; Thompson 1982) and their prey, and influences interactions with higher-order predators. Such strong tri-trophic linkages are perhaps the most significant ecological aspect of sequestration. Trophic relationships in general comprise a major pillar of community ecology (for reviews see Polis and Winemiller 1996; Morin 1999) and largely structure patterns of energy flow through communities. For predators (sensu stricto), the availability of energy has many determinants, such as mobility of prey and the capacity of predators to locate, consume, and assimilate energy from prey. Chemical defenses in plants and animals have been shown repeatedly to exert a strong influence on trophic interactions, especially in herbivorous insects and various marine invertebrates. Indeed, study of those two groups is so sophisticated that several volumes summarizing major advances have appeared (e.g., Rosenthal and Berenbaum 1991; Roitberg and Isman 1992; Meinwald and Eisner 1995; McClintock and Baker 2001; Paul et al. 2001). It is therefore surprising that similar attention to the chemical ecology of SDCs among terrestrial vertebrates has been limited to only a few well-known examples.

In principle, toxins may be sequestered at multiple trophic levels within a food chain. If, for example, the xenodontine snake *L. epinephalus* does indeed sequester toxins from dendrobatid frogs, as suggested here, such toxins would be acquired indirectly from the frogs’ invertebrate prey, some of which may in turn have acquired their toxins from a dietary source, such as plants or fungi (Saporito et al. 2012). Furthermore, geographic, seasonal, or other variation in prey availability undoubtedly influences the toxin profiles of species that sequester defensive compounds from their prey. Both spatial and temporal variation have been documented for poison frogs (Saporito et al. 2006), and geographic variation in the toxin profiles of *R. tigrinus* is suspected to reflect regional variation in toad toxins (R. A. Saporito, A. H. Savitzky, D. A. Hutchinson, and A. Mori, unpublished). A more extreme case involves a population of *R. tigrinus* that occurs on a naturally toad-free island and lacks defensive bufadienolides altogether (Hutchinson et al. 2012).

Another important aspect of sequestration is its impact on the physiology of the predator, presumably with indirect fitness consequences. For example, dendrobatid frogs exhibit unusually high aerobic scope as compared to more cryptic anurans (Pough and Taigen 1990). The active, diurnal foraging mode of aposematic dendrobatids presumably reflects their relative impunity in the face of avian predators (Saporito et al. 2007b). Additional physiological consequences of sequestration may result from the direct effects of the toxins on predators that accumulate such compounds. As noted, garter snakes (*Thamnophis*) experience a performance cost upon consumption of toxic newts, although the potential increase in predation risk is offset by the sequestration of TTX in their tissues. Consumption of toads by *R. tigrinus* results in a rapid and sustained rise in heart rate (A. H. Savitzky, D. A. Hutchinson, and A. Mori, unpublished), but the metabolic consequences of that response are not known. The species appears to have a relatively short lifespan in the wild (Fukada 1959), but whether that demographic characteristic is linked to its physiological response to bufophagy also is not known.

If bufophagy is indeed linked to reduced longevity in *R. tigrinus*, the fitness effects resulting from fewer clutches might be offset by the provisioning of eggs and offspring with bufadienolides, potentially increasing survivorship at early life history stages. Notably, recent studies have demonstrated that some dendrobatid tadpoles, once believed to lack alkaloids, in fact possess such toxins, perhaps as a result of the consumption of toxin-laden trophic eggs (R. A. Saporito, unpublished). Such transfer of toxins to larvae would extend the fitness advantage conferred by maternal sequestration to an earlier life history stage, a functional parallel to the provisioning of embryos with toxins in *Rhabdophis* (Hutchinson et al. 2008).

Although we have drawn parallels here between dietary sequestration in invertebrates and vertebrates, important differences also exist. For example, because most vertebrates exhibit relatively long lifespans and generation times in comparison to many invertebrate herbivores (Sabelis et al. 1999), vertebrates might require specific mechanisms for increasing residence times for, and tolerance of, acquired toxins in their tissues. Such complex interactions between diet, physiology, and life history remain to be explored for virtually all taxa that are known to sequester defensive toxins.

The sequestration of defensive toxins also has important implications for conservation. Although any predator will be affected negatively by a decline in its prey base, species that rely on specific prey not only for nutrients but also defensive chemicals presumably would be at even greater risk. In the case of species with aposematic coloration or conspicuous defensive behaviors, the lack of available defensive toxins would be expected to lead to a rapid increase in predation, even by predators with an evolved avoidance response. For example, if defended by sequestration of alkaloids from prey, both *Phrynosoma* and *Gastrophryne* might be especially impacted by the decline in native ant species correlated with the presence of the introduced fire ant, *Solenopsis invicta* (Stuble et al. 2009). Similarly, the precipitous decline of Neotropical anurans such as *Atelopus* (La Marca et al. 2005) may be having a negative impact on populations of *L. epinephalus* (K. Lips, personal communication), which is resistant to, and may sequester, several classes of amphibian toxins. Slug-eating snakes might be affected by declining populations of terrestrial mollusks (Lydeard et al. 2004). Furthermore, species such as *R. tigrinus*, which are adapted to store steroidal dietary toxins, may be affected disproportionately by estrogen-mimicking contaminants such as organic pesticides (Boggs et al. 2011). Clearly, a better understanding of toxin sequestration is needed if conservation biologists are to appreciate more fully the cascading trophic effects of declining prey on certain vertebrate predators.

Finally, a greater understanding of sequestered toxins in vertebrates might lead to significant biomedical insights. Many therapeutically important natural compounds presumably remain to be discovered (Zhu et al. 2011), and the mechanisms underlying resistance to accumulated toxins in sequestering species may shed light on fundamental physiological processes and on compounds of pharmacological interest. For example, the dendrobatid frog *Epipedobates bicolor* was the original source of epibatidine, a potent inhibitor of nicotinic receptors with nociceptive properties, believed to be sequestered from an unknown invertebrate source. The compound, since synthesized, has been the inspiration for several experimental drugs (Daly 2003) and has proven useful as a probe for research on nicotinic receptors (Marks et al. 2010). Similarly, the bufadienolide toxins of toads have long been known as potent cardiotonic steroids and have been employed in traditional medicines (e.g., the Chinese *chan su*; Garg et al. 2008). Snakes that tolerate and sequester bufadienolides from toads might serve as models for the investigation of certain hypertensive disorders. Advances in functional genomics and molecular modeling, recently applied to the evolution of tetrodotoxin resistance in snakes (Feldman et al. 2012), offer new opportunities to investigate the evolution of toxin resistance at the molecular level in many sequestering taxa.

## An integrated approach to the study of SDCs

We do not expect that every example described above will prove to involve sequestration of toxins. However, we believe that the abundance of circumstantial evidence, coupled with the growing number of confirmed instances of sequestration, justifies a concerted effort to pursue additional examples of SDCs among terrestrial vertebrates. We suspect that the small number of confirmed examples reflects two issues: (1) simply overlooking sequestration as a source of defensive toxins and (2) a dearth of expertise required to investigate the phenomenon. Regarding the first issue, it is our intention with this discussion to raise awareness of sequestration as a widespread phenomenon in vertebrates and to encourage researchers to consider whether additional chemically defended species might be sequestering exogenous toxins. We have been impressed with the number of times that discussions of sequestration have elicited reports of tantalizing field observations by colleagues, and we hope to inspire chemical analyses or experimentation to test the origin of defensive toxins in such cases.

The second issue is more difficult to resolve, as it requires building collaborative teams incorporating diverse expertise, especially in field biology and natural product chemistry. Indeed, the earliest and most extensive collaboration in this area, involving the alkaloid toxins of dendrobatid frogs, was the result of a fortuitous partnership between a chemist, John Daly, and herpetologist Charles Myers. Their work was stimulated by an interest in the potential pharmacological properties of integumentary secretions of amphibians (Daly 1998). Unfortunately, more than 35 years later such broadly cross-disciplinary teams remain rare, perhaps due in some measure to a decreasing emphasis on two key pillars of chemical ecology: detailed studies of natural history and natural product chemistry. Fortunately, studies of natural history are again being recognized for their central role in identifying patterns and framing questions in organismal biology and ecology (Greene 2005; Schmidly 2005; Schwenk et al. 2009). A more troubling trend is a decline in the training of natural product chemists (Eisner 2003a; Meinwald and Eisner 2003). There is particular concern for the future of basic exploratory studies of the chemical interactions among organisms (Eisner and Berenbaum 2002), studies that have sometimes been derided as “fishing expeditions”. We disagree strongly with this characterization and note a parallel to the negative perception that stigmatized biotic surveys and taxonomic revisions several decades ago. Criticized as merely descriptive, such systematic studies had, in fact, long been conducted within a context rich in implied hypotheses of phylogenetic relationships and historical effects on biogeographic patterns. We believe that many investigations of natural products, and those of SDCs in particular, similarly have been conducted within an implicit conceptual framework that addresses historical questions concerning the origin of chemical novelties and ecological questions regarding the trophic relationships underlying chemical defenses.

Just as studies of biodiversity have enjoyed a recent resurgence in interest and status, reflecting the ongoing extinction crisis and the application of new methods, studies of sequestered chemical defenses are poised to benefit from technological improvements in the analysis of small sample volumes and from a compelling conservation imperative. Extinctions appear to be accelerating among both amphibians (Collins and Crump 2009) and reptiles (Gibbons et al. 2000), unraveling the complex fabric of terrestrial food webs. Just as there is an urgent need to document the diversity of species in light of current population declines, there is a diminishing opportunity to examine the trophic interactions among species, including the intimate relationships that revolve around SDCs.

A comprehensive study of toxin sequestration should extend beyond simply determining the nature of the defensive compounds and their presence in both predator and prey, although those constitute an essential prelude to more detailed investigations. The uptake and storage of sequestered toxins must be verified by experimental studies involving either labeled or otherwise traceable compounds. Chemical analysis also can determine whether sequestered toxins are widespread in the predator’s tissues or are concentrated in specific organs. If the latter, associated changes in morphology should be explored, from subtle modifications such as enhanced vascular supply or hypertrophy of existing tissues to the origin of novel structures for storage and delivery, as in *Rhabdophis*. If morphological changes are found, a direct morphogenetic role for the sequestered compounds should be considered. Residence time and turnover for the toxins in the predator’s tissues should be examined, as should any ontogenetic changes in the concentration and chemistry of the toxins. Attention also should be paid to physiological correlates of sequestration, including the mechanism for resistance to toxins by the predator, to determine whether such resistance involves the evolution of novel physiological adaptations or simply co-option of a broad pre-existing tolerance. Both energetic and performance costs associated with either the toxins themselves or the mechanisms that confer tolerance also should be considered (Karasov and Martínez del Rio 2007). Novel behaviors or aposematic displays should be studied in taxa that sequester defensive toxins, and avoidance of such taxa by their predators should be examined, preferably by experimentation. Finally, the possibility that bioaccumulation of toxins occurs across multiple trophic levels should be considered. Such a comprehensive and integrative approach will, of course, require the participation by more than field biologists and natural product chemists. Ethologists, physiologists, evolutionary ecologists, and specialists in molecular phylogenetics and functional genomics will be needed to understand the full scope of such complex chemical interactions among species.

Whatever the outcome of individual studies, it seems likely that many new cases of sequestered defensive toxins remain to be discovered. A rich array of dietary specializations exists among amphibians and reptiles, often involving prey that are chemically defended, and little is known of the chemical interactions between such trophic specialists and their prey. Without a greater appreciation for the role that prey toxins play in the physiology, ecology, and behavior of predators, our knowledge of species interactions will be incomplete and our understanding of the forces that structure food webs will remain imperfect. By encouraging our colleagues to consider the possibility of toxin sequestration in studies of trophic relationships, we hope to stimulate additional interest in this fruitful field.
